# The Associations of Diuretics and Laxatives Use with Cardiovascular Mortality. An Individual Patient-Data Meta-analysis of Two Large Cohort Studies

**DOI:** 10.1007/s10557-019-06894-w

**Published:** 2019-08-02

**Authors:** Liesa Katharina Hoppe, Dana Clarissa Muhlack, Wolfgang Koenig, Hermann Brenner, Ben Schöttker

**Affiliations:** 1 Division of Clinical Epidemiology and Aging Research, German Cancer Research Center, Im Neuenheimer Feld 581, 69120 Heidelberg, Germany; 2 Network Aging Research, University of Heidelberg, Bergheimer Straße 20, 69115 Heidelberg, Germany; 3 Deutsches Herzzentrum München, Technische Universität München, Lazarettstraße 36, 80636 Munich, Germany; 4 DZHK (German Centre for Cardiovascular Research), partner site Munich Heart Alliance, Pettenkoferstraße 8a & 9, 80336 Munich, Germany; 5 Institute of Epidemiology and Medical Biometry, University of Ulm, Helmholtzstraße 22, 89081 Ulm, Germany

**Keywords:** Diuretics, Laxatives, Cardiovascular mortality, Epidemiology, Cohort study

## Abstract

**Purpose:**

To investigate the associations of diuretics overall, non-potassium-sparing diuretics in specific, and laxative use with cardiovascular mortality (CVM) in subjects with antihypertensive treatment.

**Methods:**

Analyses included 4253 participants, aged 50 to 75 years, from the German ESTHER cohort and 105,359 participants, aged 50 to 69 years, from the UK Biobank. Cox proportional hazard regression models were applied in both studies, and then results were pooled using random-effects model meta-analyses.

**Results:**

During 14 and 7 years of follow-up, 476 and 1616 CVM cases were observed in the ESTHER study and the UK Biobank, respectively. Compared to non-users, a 1.6-fold (hazard ratio [95% confidence interval] 1.57 [1.29; 1.90]), a 1.4-fold (1.39 [1.26; 1.53]), and no statistically significantly increased (1.13 [0.94; 1.36]) CVM were observed in users of diuretics overall, non-potassium-sparing diuretics in specific, and laxatives, respectively. Concurrent use of non-potassium-sparing diuretics and laxatives was associated with a 2-fold increased CVM (2.05 [1.55; 2.71]) when compared to users of neither diuretics nor laxatives. However, a test for interaction slightly missed statistical significance (*p* = 0.075).

**Conclusions:**

These consistent results from two large cohort studies imply that more research is needed on the safety of diuretics in routine care. Although not statistically significant in this study, a drug-drug interaction of non-potassium-sparing diuretics and laxatives appears plausible. Physicians and pharmacists are advised to clarify additional laxative use in users of non-potassium-sparing diuretics and inform about the risk of concurrent use. Moreover, closer potassium monitoring intervals (e.g., every 3 months) might be indicated in concurrent users to prevent fatal cardiovascular events.

**Electronic supplementary material:**

The online version of this article (10.1007/s10557-019-06894-w) contains supplementary material, which is available to authorized users.

## Introduction

Over-the-counter (OTC) drugs are medications that can be bought without a prescription, which consequently often happens without a physician’s advice or knowledge. Especially older individuals use OTC drugs frequently [1, 2]. In the USA, concurrent use of prescription and OTC drugs is present in 46% of community-residing individuals aged 57 to 85 years [3]. Therefore, the risk of potentially serious drug-drug interactions may be underestimated by analyses of medication claims databases, which do not record OTC drugs [4]. One prominent example are the laxatives. Approximately 20% of the elderly suffers from chronic constipation and thus uses laxatives regularly [5]. Laxatives could interact with non-potassium-sparing diuretics, which are prescribed to one in five of the elderly [6], because both drug classes can decrease serum potassium levels [7, 8]. Due to the narrow range of physiological serum potassium levels (3.5 to 5.0 mmol/L), potassium depletion may result in hypokalemia [9, 10]. Since adequate levels are of high importance for heart rhythm and function, hypokalemia can cause arrhythmias and even cardiac death [11–13].

In this context, we recently published a systematic review about the prospective association of abnormal serum potassium levels and cardiovascular mortality (CVM) [14]. In summary, the results showed that the association of hypokalemia and CVM appears to be restricted to subpopulations with hypertension or heart failure. According to guidelines, both conditions are frequently treated with diuretics [15]. Diuretic-induced hypokalemia may cause ventricular dysrythmia [16–19] which subsequently may result in cardiac death [13]. This is why hypokalemia due to use of non-potassium-sparing diuretics might explain the increased CVM in these patients. An increased cardiovascular risk by non-potassium-sparing diuretics has been shown in previous cohort studies and was explained by drug-induced hypokalemia [11, 20, 21]. However, there is a lack of studies investigating the risk of regular laxative use [22], especially in combination with non-potassium-sparing diuretics. Similarly, adverse effects of laxatives include drug-induced hypokalemia, which might consequently also result in dysrhythmia and cardiac death [23, 24].

The aim of our study is to provide the first investigation on the associations of diuretics overall, non-potassium-sparing diuretics in specific, and laxative use with CVM in subjects with antihypertensive treatment, which includes subjects with hypertension or heart failure. The drug classes were first analyzed distinctly and then jointly to detect potential drug-drug interactions. To achieve this aim, analyses were separately conducted in two large-scale cohort studies. While the German ESTHER study was used as a derivation cohort to generate hypotheses, the larger UK Biobank served as replication cohort to confirm the findings. Results from both studies were then combined in an individual patient-data meta-analysis.

## Methods

### Design and Setting

The ESTHER study (IRB approval) is an ongoing epidemiological cohort study performed in the older general population of the federal state of Saarland, Germany [25]. At baseline (2000 to 2002), 9940 men and women, aged 50 to 75 years, were recruited by their general practitioner, when presenting for a routine health check-up [26]. Data collection at baseline included physical measurements and blood sampling. Additionally, both the general practitioner and the participant provided information via detailed standardized questionnaires. Follow-up contacts were realized after 2, 5, 8, 11, and 14 years (the latter was used for the current analysis).

The UK Biobank (IRB approval) is also a general population cohort study and recruited participants aged between 40 and 69 years [27]. Recruitment of 502,616 individuals took place from 2006 to 2010 throughout the UK [28]. Data collection included a self-completed touch-screen questionnaire, a computer-assisted interview, physical and functional measures, and biological samples, as described in detail elsewhere [28]. A follow-up after 7 years was realized through linked population-level UK medical and other health-related records [28, 29].

### Mortality Ascertainment

Vital status of ESTHER participants was collected until the end of 2015 by querying the residents’ registration offices resulting in a completeness of follow-up for all-cause mortality of 99.7%. Death certificates were available from public health departments for 98.9% of ESTHER participants who had died. In the UK Biobank, almost complete mortality follow-up until 15 February 2016 was guaranteed by embedding the study within the UK’s National Health Service [27]. The primary cause of death was available for all but six UK Biobank participants (99.99% completeness).

Deaths coded with ICD-10 codes I00-I99 were considered cardiovascular deaths, hereinafter also referred to as cardiovascular mortality (CVM).

### Medication Assessment

In the ESTHER study, prescribed drugs were collected from the physician’s questionnaire. In the UK Biobank, an interview of the study participants with a trained nurse was conducted to get information on drug utilization. To define the study population of participants with antihypertensive drug treatment, the following antihypertensive drug classes (ATC codes) were used: agents acting on the renin-angiotensin system (C09), calcium channel blockers (C08), beta blocking agents (C07), diuretics (C03), and miscellaneous antihypertensive agents (C02).

Diuretics were studied in two groups: (i) non-potassium-sparing diuretics (C03AA, C03AH, C03AX, C03BA, C03BC, C03BD, C03BK, C03BX, C03CA, C03CC, and C03CX), and (ii) potassium-sparing diuretics (C03D), combinations of non-potassium-sparing diuretics with potassium (C03AB, C03BB, and C03CB), or combinations of non-potassium-sparing diuretics with potassium-sparing diuretics (C03E).

Laxative use was assessed with the following question in the participant questionnaire of the ESTHER study: “Do you at present sometimes or regularly (daily) take any of the following drugs?” A list of OTC drugs was given, with the answering options “No,” “Yes, sometimes,” and “Yes, regularly.” If study participants answered “Yes, sometimes” or “Yes, regularly” in the line asking for “laxatives” they were treated as regular users in our analysis. The UK Biobank assessed laxative use via the touchscreen questionnaire under the heading “Drugs/Medication → OTC-drugs / self-medication” with the following question: “Do you regularly (that is, most days of the week for the last 4 weeks) take any of the following? (You can select more than one answer).” Answering options included “Laxatives (e.g., Dulcolax, Senokot)” and other OTC drugs.

### Covariate Assessment

Socio-demographic characteristics and lifestyle factors were assessed as self-reported information with detailed standardized participant questionnaires in the ESTHER study and with touchscreen questionnaires in the UK Biobank.

Smoking status was assessed by questions about the participant’s past and current tobacco smoking history and finally defined via the following three categories: “never,” “previous,” or “current.” Physical activity was measured in hours of vigorous physical activity per week in the ESTHER study. Vigorous physical activity was defined as activities that cause sweating (e.g., sports and heavy physical work). Participants doing any amount of vigorous physical activity per week were defined as physically active. In the UK Biobank, physical activity was assessed as the number of days per week of at least 10 min of vigorous physical activity (defined as activities that make one sweat or breathe hard such as fast cycling, aerobics, and heavy lifting). Participants who had answered with 1 to 7 days were defined as physically active. The amounts of beverages were used to estimate grams of consumed ethanol per day and were subsequently grouped into the WHO drinking categories as follows: abstainers, category I (mild) including women with an alcohol consumption of 0–19.99 g/day or men with 0–39.99 g/day, and category II/III (moderate/heavy) including women with ≥ 20 g/day or men with ≥ 40 g/day [30].

In the ESTHER study, measurements of systolic blood pressure (SBP, in mmHg) were available from the physician’s medical conditions report of the health check-up. In the UK Biobank, SBP measurements were conducted by automated reading at the left upper arm (range returned by the Omron device is 0–255 mmHg). Body mass index (in kg/m^2^) was calculated based on weight (kg) and height (m) and categorized according to a slightly modified version of the WHO standards as follows: < 25, 25 to < 30, and ≥ 30 kg/m^2^ [31]. Potential kidney damage was defined by urinary albumin levels ≥ 20 mg/L. In the ESTHER study, urinary albumin was measured by nephelometry with the BN II system using OSAL N antiserum against albumin (both Siemens, Marburg, Germany). In the UK Biobank, urinary albumin was determined with immunoturbidimetry on a Beckman Coulter AU5400 (Brea, USA).

Information on diseases (diabetes mellitus, heart failure, and coronary heart disease (CHD)) and a history of cardiovascular events (myocardial infarction (MI) and stroke) were based on physician-reported information in the ESTHER study and on self-reported information from a verbal interview in the UK Biobank. To identify subjects with diabetes mellitus, additionally reported information on antidiabetic drugs was used (physician-reported in the ESTHER study and self-reported in the UK Biobank). CHD was defined as a composite of angina pectoris and MI. The heart failure prevalence in the UK Biobank was implausibly low (0.03%) most probably because of self-reporting and was therefore not used in the analyses.

Anticholinergic drug use included use of drugs classified as having a moderate (score 2) or severe (score 3) anticholinergic potential according to the anticholinergic cognitive burden scoring [32–34]. Opioid users were identified via the ATC codes N02A (opioids) and N07BC (drugs used in opioid dependence).

### Statistical Analysis

To have a more comparable baseline age in the two studies, we excluded 117,894 participants younger than 50 years from the total UK Biobank sample of *n* = 502,616. Furthermore, in order to have a comparable baseline cardiovascular risk of diuretics users and a control group, only users of antihypertensive drugs were included in the current analysis (*n*_excluded_ = 279,348). Further 15 participants were excluded, as their causes of death were unknown (ICD-10 code R98, R99) or missing. Finally, the analytical sample size for the UK Biobank was *n* = 105,359. Likewise, we excluded from the 9940 ESTHER baseline participants those with no antihypertensive treatment (*n*_excluded_ = 5622), an unknown cause of death (*n*_excluded_ = 56), or loss to follow-up (*n*_excluded_ = 9), leaving an analytical sample size of *n* = 4253.

Exposure to diuretics and laxatives was assessed in two ways: distinctly and jointly. In distinct analyses, laxatives and diuretics were put separately into models. Furthermore, we analyzed diuretics use overall and more specifically sub-divided into two groups:

1. Users of non-potassium-sparing diuretics
2. Users of potassium-sparing diuretics/combinations of non-potassium-sparing diuretics with potassium or potassium-sparing diuretics.

**Fig. 1:**
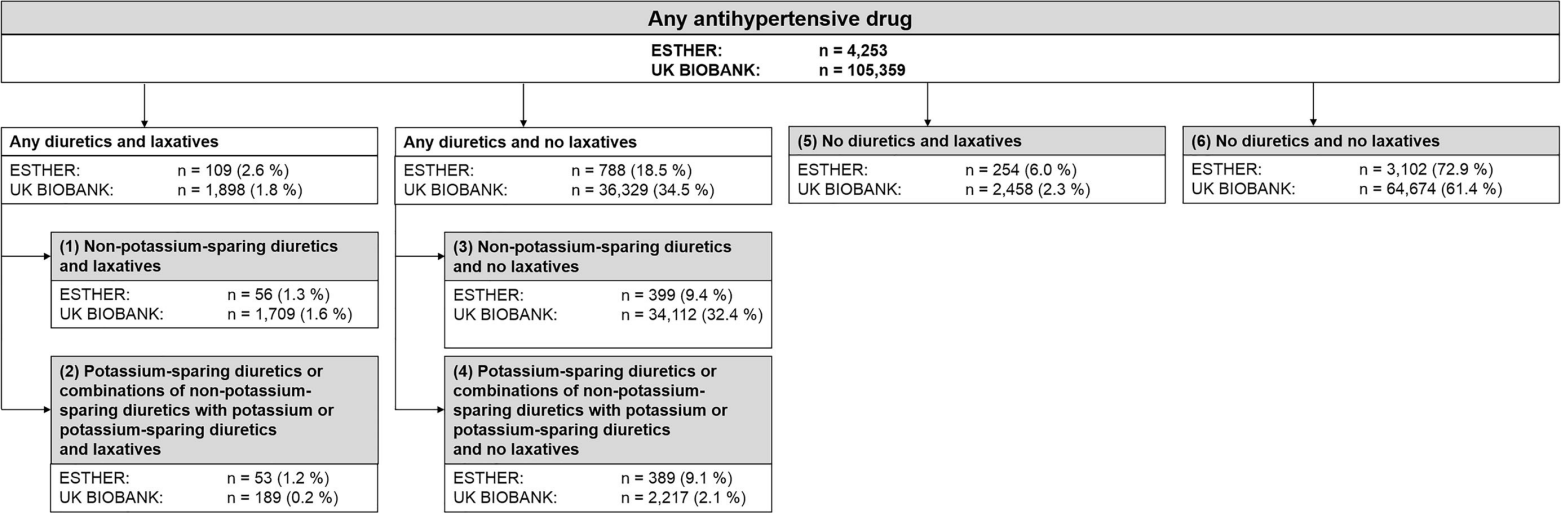
Flow chart showing the categorization of the study participants into six mutually exclusive treatment groups within the ESTHER study and the UK Biobank

In a sensitivity analysis conducted in the large UK Biobank only, the second group was further divided into its three subgroups. For joint analyses of concurrent diuretics and laxative use, participants were allocated to six mutually exclusive treatment groups (Fig. 1):

1. Non-potassium-sparing diuretics and laxatives
2. Non-potassium-sparing diuretics and no laxatives
3. Potassium-sparing diuretics/combinations of non-potassium-sparing diuretics with potassium or potassium-sparing diuretics and laxatives
4. Potassium-sparing diuretics/combinations of non-potassium-sparing diuretics with potassium or potassium-sparing diuretics and no laxatives
5. No diuretics and laxatives
6. No diuretics and no laxatives.

Differences in baseline characteristics in selected drug user groups were assessed with *χ*^2^ tests. The associations of the aforementioned drug exposure groups with CVM were assessed with Cox proportional hazard regression models to estimate HRs and 95%CIs. All analyses were adjusted for age, sex, smoking status, physical activity, alcohol consumption (assessed in categories of the WHO [30]), SBP, BMI, potential kidney damage, diabetes mellitus, heart failure (in the ESTHER study only), CHD, history of MI, history of stroke, anticholinergic drug use, and use of opioids. We adjusted for the latter two drug classes because they can cause chronic constipation and are known to be associated with mortality [35, 36]. Age and SBP were modeled continuously and all other co-variables with the categorizations shown in Table 1. Subgroup analyses were conducted for age (≥ 65 years vs. < 65 years), sex (men vs. women), potential kidney damage (urinary albumin ≥ 20 mg/L vs. < 20 mg/L), CHD (yes vs. no), and heart failure (yes vs. no).

**Table 1:** Baseline characteristics of the analyzed participants with antihypertensive treatment of the ESTHER study (Germany, baseline 2000–2002) and the UK Biobank (UK, baseline 2006–2010)

	ESTHER (*n* = 4253)			UK Biobank (*n* = 105,359)		
Characteristics	*n*_total_^a^	*n*_char_ (%)	Mean (SD)	*n*_total_^a^	*n*_char_ (%)	Mean (SD)
Age (years)	4253		64 (6)	105,359		62 (5)
Age ≥ 65 years	4253	2024 (47.6)		105,359	38,203 (36.3)	
Sex (male)	4253	1917 (45.1)		105,359	55,751 (52.9)	
Smoking	4128			104,621		
Never		2136 (51.7)			50,280 (48.1)	
Former		1440 (34.9)			45,054 (43.1)	
Current		552 (13.4)			9287 (8.9)	
Vigorous physical activity^b^	4240			96,972		
No		2455 (57.9)			44,401 (45.8)	
Yes		1785 (42.1)			52,571 (54.2)	
Alcohol consumption^c^	3803			88,913		
Abstainer		1381 (36.3)			10,804 (12.2)	
WHO category I		2196 (57.7)			55,816 (62.8)	
WHO category II/III		226 (5.9)			22,293 (25.1)	
SBP (mmHg)	4246		145 (20)	98,147		147 (19)
BMI (kg/m^2^)	4253		28.9 (4.5)	104,683		29.5 (5.2)
< 25		771 (18.1)			18,823 (18.0)	
25 to < 30		1996 (47.0)			44,376 (42.4)	
≥ 30		1483 (34.9)			41,484 (39.6)	
Urinary albumin (mg /L)	4220			41,488		
< 20		3303 (78.3)			27,495 (66.3)	
≥ 20		917 (21.7)			13,993 (33.7)	
Diabetes mellitus	4192	944 (22.5)		105,359	11,375 (10.8)	
Heart failure	4206	748 (17.8)		104,935	N.A.^d^	
CHD	4252	946 (22.3)		104,935	17,627 (16.8)	
History of MI	4079	417 (10.2)		104,935	9345 (8.9)	
History of stroke	4066	222 (5.5)		104,935	2940 (2.8)	
Anticholinergic drug use	4253	224 (5.3)		105,359	7160 (6.8)	
Opioid use	4253	35 (0.8)		105,359	9615 (9.1)	
Laxative use	4253	352 (8.2)		105,359	4322 (4.1)	
Diuretics use	4253	897 (21.1)		105,359	38,227 (36.3)	
Non-potassium-sparing diuretics		455 (10.7)			35,821 (34.0)	
Potassium-sparing diuretics or combinations^e^		442 (10.4)			2406 (2.3)	

*BMI* body mass index, *CHD* coronary heart disease, *MI* myocardial infarction, *N.A.* not applicable, *n*_*char*_ number of participants with the characteristics, *n*_*total*_ number of participants with data for the characteristic, *SBP* systolic blood pressure, *SD* standard deviation, *UK* United Kingdom, *WHO* World Health Organization

^a^Does not always add up to the total study sizes (*n*) due to missing values

^b^Vigorous physical activity was measured in hours per week (ESTHER) or in number of days per week of at least 10 min of activity (UK Biobank): “No”: Participants not doing any amount of vigorous physical activity, “Yes”: Participants doing any amount of vigorous physical activity

^c^Alcohol consumption in categories of the WHO ^30^: Category I including women with an alcohol consumption of 0–19.99 g/day or men with 0–39.99 g/day, category II including women with an alcohol consumption of 20–39.99 g/day or men with 40–59.99 g/day, and category III including women with an alcohol consumption of ≥ 40 g/day or men with ≥ 60 g/day

^d^Not sufficiently assessed in the UK Biobank (unreliable self-report) and therefore not applicable for use

^e^Group comprises users of potassium-sparing diuretics/combinations of non-potassium-sparing diuretics with potassium or potassium-sparing diuretics

Results of both studies were combined by random-effects model meta-analyses using the software Comprehensive Meta-Analysis 2.0 (Biostat). In a sensitivity analysis, the follow-up time of the ESTHER study (14 years) was restricted to the follow-up time of the UK Biobank (7 years) to check whether the different lengths of follow-up importantly influenced the results. All analyses were performed with SAS 9.4. Missing values were imputed using the MCMC algorithm of the SAS procedure PROC MI. Five imputed datasets were created, and analyses of these datasets were combined by the SAS procedure PROC MIANALYZE. All statistical tests were two-sided with an *α*-level of 0.05.

## Results

### Characteristics of the Study Population

Baseline characteristics of antihypertensive medication users of both studies are shown in Table 1. The mean age of the 4253 ESTHER participants and the 105,359 UK Biobank participants were 64 years and 62 years, respectively. There was a higher proportion of men in the UK Biobank (52.9%) than in the ESTHER study (45.1%). Furthermore, the proportions of physically active participants and moderate/heavy drinkers (WHO category II/III) were higher in the UK Biobank. The prevalence of diseases and cardiac events was higher in the ESTHER study, while elevated urinary albumin levels (≥ 20 mg/L) were more prevalent in the UK Biobank indicating a higher proportion of participants with potential kidney damage in this cohort.

Further, Table 1 shows that self-reported, regular laxative use was higher in the ESTHER study (8.2%) than in the UK Biobank (4.1%). One out of five ESTHER participants used diuretics (21.1%), whereas in the UK Biobank, more than every third participant did (36.3%). The difference can mostly be explained by lower β-blocker use in the UK Biobank (30.6%) compared to 49.3% in the ESTHER study (Table S1). All other antihypertensive drug classes (calcium channel blockers, angiotensin-converting enzyme inhibitors, and angiotensin receptor blockers) were similarly frequently used (Table S1). While the diuretics users in the ESTHER study (*n* = 897) consisted of equal parts of non-potassium-sparing diuretics users (50.7%) and of potassium-sparing diuretics/combinations of non-potassium-sparing diuretics with potassium or potassium-sparing diuretics users (49.3%), the diuretics users in the UK Biobank (*n* = 38,227) mainly took non-potassium-sparing diuretics (93.7%), and only few (6.3%) used potassium-sparing diuretics/combinations of non-potassium-sparing diuretics with potassium or potassium-sparing diuretics (Table 1).

Population characteristics that were statistically significantly associated with regular laxative use in both studies were age ≥ 65 years, female sex, mild alcohol consumption (category I), history of stroke, anticholinergic drug use, opioid use, and diuretics use (Table S2). Use of diuretics was statistically significantly positively associated with older age (≥ 65 years), female sex, lower alcohol consumption, higher body mass index (BMI), history of stroke, opioid use, and laxative use in both cohorts (Table S3). Comparing more specifically users of non-potassium-sparing diuretics and users of potassium-sparing diuretics/combinations of non-potassium-sparing diuretics with potassium or potassium-sparing diuretics, most characteristics were comparable (Table S4). Interestingly, subjects with potassium-sparing diuretics/combinations of non-potassium-sparing diuretics with potassium or potassium-sparing diuretics more frequently had blood pressure < 140 mmHg in both cohorts. However, a different pattern in the two studies was observed for the cardiovascular disease burden. Whereas CHD and a history of MI in users of non-potassium-sparing diuretics were more prevalent in the ESTHER study, these cardiovascular diseases were statistically significantly less prevalent in the UK Biobank.

### Associations of Laxatives and Diuretics Use with CVM in Distinct Analyses

During a median follow-up time of 14 years, 476 cardiovascular deaths were observed in the ESTHER study (rate per 1000 person-years 8.7). In the UK Biobank, 1616 cardiovascular deaths were detected during a median follow-up of 7 years (rate per 1000 person-years 2.2). Hazard ratios (HRs) and 95% confidence intervals (95%CIs) for the associations of laxatives and diuretics use with CVM are shown in Table 2.

**Table 2:** Associations with CVM comparing users and non-users of laxatives, diuretics overall, and diuretics in specific in the ESTHER study (Germany, baseline 2000–2002, mean baseline age 64 years, 14 years of mortality follow-up), in the UK Biobank (UK, baseline 2006–2010, mean baseline age 62 years, 7 years of mortality follow-up), and in a meta-analysis of the two studies

Drug class	ESTHER (*n* = 4253; 476 cases)			UK Biobank (*n* = 105,359; 1616 cases)			Meta-analysis (*n* = 109,612; 2092 cases)		
	*n*^a^	*n*_cases_	HR (95%CI)^b^	*n*^a^	*n*_cases_	HR (95%CI)^b^	*n*^a^	*n*_cases_	HR (95%CI)^c^
Laxatives									
Non-users	3890	433	Ref.	101,003	1526	Ref.	104,893	1959	Ref.
Users	363	43	0.99 (0.70; 1.41)	4356	90	1.19 (0.96; 1.48)	4719	133	1.13 (0.94; 1.36)
Diuretics overall									
Non-users	3356	327	Ref.	67,132	887	Ref.	70,488	1214	Ref.
Users	897	149	*1.39 (1.13; 1.70)*	38,227	729	*1.70 (1.53; 1.88)*	39,124	878	*1.57 (1.29; 1.90)*
Diuretics in specific									
Non-users	3356	327	Ref.	67,132	887	Ref.	70,448	1214	Ref.
Users of non-potassium-sparing diuretics	455	90	*1.49 (1.17; 1.89)*	35,821	597	*1.37 (1.23; 1.52)*	36,276	687	*1.39 (1.26; 1.53)*
Users of potassium-sparing diuretics or combinations^d^	442	59	1.10 (0.83; 1.46)	2406	132	*3.03 (2.52; 3.64)*	2848	191	1.84 (0.68; 4.96)

Italicized entries are statistically significant (*ρ* < 0.05)

*CI* confidence interval, *CVM* cardiovascular mortality, *HR* hazard ratio, *Ref*. reference, *UK* United Kingdom

^a^Sample sizes exemplarily taken from imputed data set no. 1

^b^Adjusted for age, sex, smoking status, physical activity, alcohol consumption, systolic blood pressure, body mass index, potential kidney damage (urinary albumin ≥ 20 mg/L), diabetes mellitus, heart failure (in ESTHER study only), coronary heart disease, history of myocardial infarction, history of stroke, anticholinergic drug use, and use of opioids

^c^Results of the two studies combined by random-effects model meta-analysis

^d^Group comprises users of potassium-sparing diuretics/combinations of non-potassium-sparing diuretics with potassium or potassium-sparing diuretics

#### Laxatives

In both studies, no statistically significantly increased CVM was observed in laxative users compared to non-users. The association of laxative use and CVM was also not statistically significant when combining the results of both studies by random-effects model meta-analysis (HR [95%CI] 1.13 [0.94; 1.36]).

#### Diuretics

A statistically significantly increased CVM in users of diuretics overall was observed in both studies. The corresponding pooled effect estimate revealed a 1.6-fold increased CVM of diuretics users compared to non-users, who used other antihypertensive drugs (HR [95%CI] 1.57 [1.29; 1.90]). Furthermore, there was a statistically significant association of non-potassium-sparing diuretics use and CVM in both studies and the pooled HR [95%CI] was 1.39 [1.26; 1.53]. Results of the ESTHER study and UK Biobank diverged for users of potassium-sparing diuretics/combinations of non-potassium-sparing diuretics with potassium or potassium-sparing diuretics, and when pooling these results of both studies by random-effects model meta-analysis, the HR point estimate was not statistically significant (HR [95%CI] 1.84 [0.68; 4.96]). The strong, statistically significant association for this group in the UK Biobank (HR [95%CI] 3.03 [2.52; 3.64]) was based on both subjects taking potassium-sparing diuretics only and subjects using potassium-sparing diuretics in combination with non-potassium-sparing diuretics (Table S5). Users of a combination of non-potassium-sparing diuretics and potassium had no statistically significantly increased CVM (Table S5).

#### Subgroup Analyses

The above-mentioned associations of laxatives and diuretics use with CVM were further assessed in subgroups by age, sex, urinary albumin levels, CHD, and heart failure (Table 3). The subgroup analysis for heart failure was only carried out in the ESTHER study because of an insufficient heart failure assessment in the UK Biobank (unreliable self-reports).

**Table 3:** Associations with CVM comparing users and non-users of laxatives, diuretics overall, and diuretics in specific assessed in subgroups by age, sex, urinary albumin levels, and heart failure (the latter only in the ESTHER study)

		ESTHER (n = 4253; ***n***_cases_ = 476)			UK Biobank (n = 105,359; n_cases_ = 1616)			Meta-analysis (***n*** = 109,612; ***n***_cases_ = 2092)		
Characteristic	Subgroup	***n***^a^	***n***_cases_	HR (95%CI)^b^	***n***^a^	***n***_cases_	HR (95%CI)^b^	***n***^a^	***n***_cases_	HR (95%CI)^c^
Users of laxatives compared to non-users of laxatives										
Age (years)	< 65	2229	126	0.94 (0.46; 1.92)	67,156	818	*1.55 (1.16; 2.06)*	69,385	944	1.35 (0.87; 2.09)
	≥ 65	2024	350	0.97 (0.65; 1.46)	38,203	798	0.88 (0.63; 1.25)	40,227	1148	0.92 (0.71; 1.19)
Sex	Female	2336	207	0.93 (0.56; 1.52)	49,608	365	1.11 (0.77, 1.60)	51,944	572	1.04 (0.78; 1.40)
	Male	1917	269	1.09 (0.66; 1.78)	55,751	1251	1.23 (0.93, 1.62)	57,668	1520	1.20 (0.94; 1.52)
Albumin (mg/L)	< 20	3328	316	1.02 (0.68; 1.54)	64,433	820	1.25 (0.90; 1.74)	67,761	1136	1.15 (0.89; 1.49)
	≥ 20	925	160	0.90 (0.48; 1.69)	40,926	796	1.13 (0.80; 1.60)	41,851	956	1.07 (0.79; 1.45)
CHD	No	3307	304	1.07 (0.70; 1.64)	87,731	967	1.23 (0.91; 1.66)	91,038	1271	1.17 (0.92; 1.50)
	Yes	946	172	0.82 (0.47; 1.45)	17,628	649	1.17 (0.84; 1.62)	18,574	821	1.06 (0.77; 1.45)
Heart failure	No	3489	326	1.07 (0.71; 1.61)	–	–	–	–	–	–
	Yes	764	150	0.83 (0.46; 1.49)	–	–	–	–	–	–
Users of diuretics overall compared to non-users of diuretics										
Age (years)	< 65	2229	126	*1.52 (1.01; 2.29)*	67,156	818	*1.69 (1.46; 1.95)*	69,385	944	*1.67 (1.46; 1.91)*
	≥ 65	2024	350	*1.38 (1.09; 1.75)*	38,203	798	*1.71 (1.48; 1.97)*	40,227	1148	*1.57 (1.28; 1.93)*
Sex	Female	2336	207	1.20 (0.88; 1.64)	49,608	365	*1.34 (1.09; 1.66)*	51,944	572	*1.29 (1.09; 1.54)*
	Male	1917	269	*1.55 (1.18; 2.03)*	55,751	1251	*1.84 (1.64; 2.07)*	57,668	1520	*1.77 (1.53; 2.04)*
Albumin (mg/L)	< 20	3328	316	*1.33 (1.03; 1.72)*	64,433	820	*1.68 (1.41; 2.00)*	67,761	1136	*1.52 (1.22; 1.91)*
	≥ 20	925	160	*1.53 (1.08; 2.17)*	40,926	796	*1.72 (1.46; 2.03)*	41,851	956	*1.68 (1.45; 1.95)*
CHD	No	3307	304	1.11 (0.84; 1.46)	87,731	967	*1.33 (1.17; 1.51)*	91,038	1271	*1.27 (1.09; 1.48)*
	Yes	946	172	*1.85 (1.36; 2.54)*	17,628	649	*2.53 (2.15; 2.98)*	18,574	821	*2.23 (1.65; 3.01)*
Heart failure	No	3489	326	1.08 (0.82; 1.41)	–	–	–	–	–	–
	Yes	764	150	*2.15 (1.53; 3.02)*	–	–	–	–	–	–
		ESTHER (***n*** = 3811; ***n***_cases_ = 417)			UK Biobank (***n*** = 102,953; ***n***_cases_ = 1484)			Meta-analysis (***n*** = 106,764; ***n***_cases_ = 1901)		
Users of non-potassium-sparing diuretics compared to non-users of diuretics										
Age (years)	< 65	2030	115	*2.01 (1.24; 3.24)*	65,722	752	*1.48 (1.27; 1.72)*	67,752	867	*1.58 (1.24; 2.02)*
	≥ 65	1781	302	*1.48 (1.11; 1.97)*	37,231	732	*1.53 (1.32; 1.78)*	39,012	1034	*1.52 (1.33; 1.74)*
Sex	Female	2058	182	*1.46 (1.00; 2.12)*	48,188	339	*1.26 (1.02; 1.57)*	50,246	521	*1.31 (1.08; 1.58)*
	Male	1753	235	*1.63 (1.17; 2.28)*	54,765	1145	*1.61 (1.42; 1.82)*	56,518	1380	*1.61 (1.44; 1.81)*
Albumin (mg/L)	< 20	2977	272	*1.47 (1.07; 2.03)*	62,978	750	*1.47 (1.22; 1.76)*	65,955	1022	*1.47 (1.25; 1.72)*
	≥ 20	834	145	*1.84 (1.24; 2.73)*	39,975	734	*1.55 (1.30; 1.84)*	40,809	879	*1.59 (1.36; 1.87)*
CHD	No	2985	270	1.15 (0.81; 1.64)	85,895	908	*1.21 (1.06; 1.39)*	88,880	1178	*1.20 (1.06; 1.36)*
	Yes	826	147	*2.27 (1.58; 3.27)*	17,058	576	*2.22 (1.87; 2.65)*	17,884	723	*2.23 (1.91; 2.61)*
Heart failure	No	3161	294	1.14 (0.80; 1.61)	–	–	–	–	–	–
	Yes	650	123	*2.60 (1.76; 3.84)*	–	–	–	–	–	–
		ESTHER (***n*** = 3798; ***n***_cases_ = 386)			UK Biobank (***n*** = 69,538; ***n***_cases_ = 1019)			Meta-analysis (***n*** = 73,336; ***n***_cases_ = 1405)		
Users of potassium-sparing diuretics or diuretics combinations^d^ compared to non-users of diuretics										
Age (years)	< 65	2039	103	1.03 (0.54; 1.95)	45,221	532	*3.68 (2.82; 4.82)*	47,260	635	2.02 (0.58; 7.01)
	≥ 65	1759	283	1.24 (0.90; 1.72)	24,317	487	*3.51 (2.67; 4.60)*	26,076	770	2.09 (0.76; 5.81)
Sex	Female	2080	167	0.92 (0.59; 1.43)	29,134	203	*2.22 (1.45; 3.39)*	31,214	370	1.43 (0.60; 3.40)
	Male	1718	219	*1.47 (1.01; 2.14)*	40,404	816	*1.40 (1.10; 1.78)*	42,122	1035	*1.42 (1.16; 1.74)*
Albumin (mg/L)	< 20	3007	264	1.16 (0.83; 1.63)	42,145	530	*3.91 (2.95; 5.19)*	45,152	794	2.14 (0.65; 7.03)
	≥ 20	791	122	1.13 (0.65; 1.96)	27,393	489	*3.37 (2.52; 4.51)*	28,184	611	2.00 (0.69; 5.84)
CHD	No	3012	262	1.03 (0.71; 1.50)	55,521	593	*3.28 (2.49; 4.33)*	58,533	855	1.85 (0.60; 5.76)
	Yes	786	124	1.39 (0.88; 2.18)	14,017	426	*3.95 (3.02; 5.18)*	14,803	550	2.38 (0.86; 6.63)
Heart failure	No	3178	285	1.01 (0.70; 1.46)	–	–	–	–	–	–
	Yes	620	101	*1.61 (1.00; 2.59)*	–	–	–	–	–	–

Italicized entries are statistically significant (*ρ* < 0.05)

*CHD* coronary heart disease, *CI* confidence interval, *CVM* cardiovascular mortality, *HR* hazard ratio

^a^Sample sizes exemplarily taken from imputed data set no. 1

^b^Adjusted for age, sex, smoking status, physical activity, alcohol consumption, systolic blood pressure, body mass index, potential kidney damage (urinary albumin ≥ 20 mg/L), diabetes mellitus, heart failure (in ESTHER study only), coronary heart disease, history of myocardial infarction, history of stroke, anticholinergic drug use, and use of opioids

^c^Results of the two studies combined by random-effects model meta-analysis

^d^Group comprises users of potassium-sparing diuretics/combinations of non-potassium-sparing diuretics with potassium or potassium-sparing diuretics

Moreover, no association of laxative use and CVM was observed in all subgroups after pooling the two studies by meta-analyses. In analyses on diuretics, participants aged 65 and older as well as participants with potential kidney damage (indicated by urinary albumin levels ≥ 20 mg/L) did not have substantially stronger associations of diuretics use and CVM. However, males, subjects with CHD, and subjects with heart failure had substantially stronger associations with CVM in all diuretics analyses with only one exception (sex-stratified analysis in the UK Biobank for users of potassium-sparing diuretics/combinations of non-potassium-sparing diuretics with potassium or potassium-sparing diuretics).

### Association of Laxatives and Diuretics Use with CVM in Joint Analyses

Figure 1 shows the categorization of the study participants into six mutually exclusive treatment groups of possible combinations of non-potassium-sparing diuretics, potassium-sparing diuretics/combinations of non-potassium-sparing diuretics with potassium or potassium-sparing diuretics, and laxatives. Concurrent use of non-potassium-sparing diuretics and laxatives was comparably rare in both cohorts (56 participants (1.3%) in the ESTHER study and 1709 participants (1.6%) in the UK Biobank).

The associations of the mutually exclusive treatment groups with CVM, using study participants taking neither diuretics nor laxatives (group 6) as the control group, are presented in Table 4. Concurrent users of non-potassium-sparing diuretics and laxatives had a higher CVM in both studies (HR [95%CI] for meta-analysis 2.05 [1.55; 2.71]) than those who used only non-potassium-sparing diuretics (HR [95%CI] for meta-analysis 1.50 [1.36; 1.66]), which speaks for a drug-drug interaction. However, the difference between these groups was not statistically significant. Similarly, a test for interaction of the variables “non-potassium-sparing diuretics use” and “laxative use” was not statistically significant either (*β*, *p* for meta-analysis + 0.718, 0.075) but not far from the cut-off for statistical significance (*p* = 0.05). Additional laxative use of subjects in the group “users of potassium-sparing diuretics/combinations of non-potassium-sparing diuretics with potassium or potassium-sparing diuretics” resulted in lower CVM (HR [95%CI] for meta-analysis 1.43 [0.33; 6.22]) than no additional laxative use (HR [95%CI] for meta-analysis 2.19 [0.79; 6.08]). Although confidence intervals overlapped widely, the *p* value for an interaction test was again not far from a statistically significant finding (*β*, *p* for meta-analysis − 0.580, 0.076). The different directions of the coefficients for the two interaction terms are biologically plausible because they suggest an additional risk by comparing laxatives and non-potassium-sparing diuretics and a protective effect by comparing laxatives and potassium-sparing diuretics/combinations of non-potassium-sparing diuretics with potassium or potassium-sparing diuretics. In the latter group, the hypokalemic effects of laxatives may counteract diuretic-induced hyperkalemia.

**Table 4:** Associations with CVM in mutually exclusive treatment groups in the ESTHER study (Germany, baseline 2000–2002, mean age 64 years) and the UK Biobank (UK, baseline 2006–2010, mean age 62 years)

	Esther (*n* = 4253; 476 cases)			UK Biobank (*n* = 105,359; 1616 cases)			Meta-analysis (*n* = 109,612; 2092 cases)		
Treatment group	*n*^a^	*n*_cases_ (%)	HR (95%CI)^b^	*n*^a^	*n*_cases_ (%)	HR (95%CI)^b^	*n*^a^	*n*_cases_ (%)	HR (95%CI)^c^
Non-potassium-sparing diuretics and laxatives	56	15 (26.8)	*2.26 (1.31; 3.90)*	1709	39 (2.3)	*1.98 (1.43; 2.75)*	1765	54 (3.1)	*2.05 (1.55; 2.71)*
Non-potassium-sparing diuretics and no laxatives	399	75 (18.8)	*1.44 (1.10; 1.87)*	34,112	558 (1.6)	*1.51 (1.35; 1.68)*	34,511	633 (1.8)	*1.50 (1.36; 1.66)*
Potassium-sparing diuretics or combinations^d^ and laxatives	53	3 (5.7)	0.59 (0.15; 2.33)	189	9 (4.8)	*2.70 (1.39; 5.23)*	242	12 (5.0)	1.43 (0.33; 6.22)
Potassium-sparing diuretics or combinations^d^ and no laxatives	389	56 (14.4)	1.29 (0.95; 1.75)	2217	123 (5.6)	*3.66 (3.01; 4.44)*	2606	179 (6.9)	2.19 (0.79; 6.08)
No diuretics and laxatives	254	25 (9.8)	0.93 (0.61; 1.42)	2458	42 (1.7)	1.15 (0.84; 1.58)	2712	67 (2.5)	1.07 (0.83; 1.37)
No diuretics and no laxatives	3102	302 (9.7)	Ref.	64,674	845 (1.3)	Ref.	67,776	1147 (1.7)	Ref.

Italicized entries are statistically significant (*ρ* < 0.05)

*CI* confidence interval, *CVM* cardiovascular mortality, *HR* hazard ratio, *Ref.* reference, *UK* United Kingdom

^a^Sample sizes exemplarily taken from imputed data set no. 1

^b^Adjusted for age, sex, smoking status, physical activity, alcohol consumption, systolic blood pressure, body mass index, potential kidney damage (urinary albumin ≥ 20 mg/L), diabetes mellitus, heart failure (in ESTHER study only), coronary heart disease, history of myocardial infarction, history of stroke, anticholinergic drug use, and use of opioids

^c^Results of the two studies combined by random-effects model meta-analysis

^d^Group comprises users of potassium-sparing diuretics/combinations of non-potassium-sparing diuretics with potassium or potassium-sparing diuretics

### Sensitivity Analysis

In a sensitivity analysis using only data from the first 7 years of follow-up of the ESTHER study, the effect estimates were comparable or slightly stronger than those from the analysis with the complete follow-up time of 14 years (Table S6).

## Discussion

In this meta-analysis of elderly users of antihypertensive drugs from two large cohort studies, use of diuretics overall, but not regular use of laxatives, was associated with CVM. Subgroup analyses suggested a particularly strongly increased CVM in users of diuretics overall who were male, and had CHD or heart failure. There were no statistically significant differences among the specific diuretics classes in the results of the meta-analyses. However, signs for a potential drug-drug interaction of non-potassium-sparing diuretics and concurrent regular laxative use were observed, but tests for interaction were not statistically significant.

### Discussion of the Results

#### Association of Laxative Use with CVM

This is the first observational analysis on the cardiovascular risk of regular laxative use in a European population. Regular laxative use was not associated with CVM in any of the analyses. This did not support our hypothesis that hypokalemia by regular laxative use [23, 24] may result in an increased CVM [37, 38] in consequence of hypokalemia-induced ventricular arrhythmias [39]. Several aspects can explain the failure to confirm this hypothesis. First, most laxative users may have taken laxatives for a long time and tolerated them well without developing arrhythmias (prevalent users). Individuals with very high laxative use, who are prone to hypokalemia-induced ventricular arrhythmias, may have died before study initiation. This phenomenon known as healthy-user/sick-stopper bias is common in studies with a prevalent user design [35] and may have biased our result towards a null association. Second, discontinuation of laxative use could have happened during follow-up. Those study participants, however, remained assigned to the user group in our analysis and could have attenuated the effect estimate for the exposure group towards a null association. Third, our definition of “regular laxative use” included users that take laxatives “sometimes” (ESTHER) or “on most of the days of the month” (UK Biobank). While the definition of the UK Biobank seems to be appropriate to define regular use, the definition of the ESTHER study is less clear and could also include some individuals that take laxatives rarely, which could have biased the effect estimate towards the null. Fourth, both studies did not ask for the names of the OTC drugs used, and therefore laxatives that do not cause hypokalemia (e.g., the bulk-forming laxatives linseed or psylla seeds) could not be excluded. Therefore, more studies on laxative use and CVM are needed. These studies should preferably have a new user design (with start of follow-up at the first initiation of drug exposure), define regular laxative use as use of laxatives that can cause hypokalemia on most days of the month, and include repeated assessments of regular laxative use.”

#### Association of Diuretics Use with CVM

With respect to non-potassium-sparing diuretics, we observed a 1.4-fold increased CVM in the meta-analysis of the results from the two studies. This is in line with the results of Cooper et al. [21] (population: patients with left ventricular dysfunction; outcome: arrhythmic death), Ahmed et al. [20] (population: heart failure patients; outcome: long-term mortality), and Alharbi et al. [40] (population: cases with cardiac arrest and controls from the general population; outcome: cardiac arrest). However, our results are not directly comparable to these studies because we included a population with a lower cardiovascular risk (e.g., a mix of patients with hypertension and/or heart failure receiving antihypertensive drugs).

However, a network meta-analysis of clinical trials did not show higher cardiovascular risks of low-dose diuretics as first-line antihypertensive treatment compared to β-blockers, angiotensin-converting enzyme inhibitors, calcium channel blockers, alpha-blockers, and angiotensin receptor blockers; rather the opposite was observed [41]. The divergent results can have several reasons: the efficacy-effectiveness gap of clinical trials [42], non-comparable study populations, or an insufficient control for confounding in observational studies. The last point is supported by the fact that our analysis was limited by insufficient control for confounders, such as heart failure and CHD both presenting a higher baseline cardiovascular risk for the affected patients. In practice, diuretics are often prescribed in combinations with other antihypertensive drug classes for subjects with high cardiovascular risk or a blood pressure that cannot be controlled by one agent [15]. An intensive blood pressure control with two or more antihypertensive drugs is particularly important in subjects with a history of MI or other cardiovascular events [15, 43]. In addition, loop diuretics are most often used as part of the guideline treatment in symptomatic heart failure patients (NYHA class II to IV) [44]. Therefore, it was not surprising that our analyses showed that diuretics users more frequently had heart failure, CHD, and histories of MI and stroke compared to non-users (Table S3).

Consequently, a better adjustment for heart failure and CHD would have been desirable, but NYHA classification and CHD severity were not available in the two studies. Therefore, the particularly strong increased CVM in diuretics users with CHD or heart failure should be interpreted with caution. This strong association might be rather due to the fact that individuals receiving diuretics probably had more severe stages of CHD or heart failure than non-users. Consequently, further observational studies are needed with detailed information on these two diseases to corroborate our results.

Finally, because poor renal function is associated with hyperkalemia [45] and increased CVM [46, 47], it was of interest whether results for diuretics use differed according to kidney function. However, diuretics users with potential kidney damage (indicated by urinary albumin levels ≥ 20 mg/L) did not show stronger associations with CVM. An explanation may be that we mainly focused on non-potassium-sparing diuretics users, who are rather prone to hypokalemia than to hyperkalemia. The group with a possible risk of hyperkalemia (potassium-sparing diuretics users), however, was not separately investigated in subgroups by urinary albumin.

#### Potential Drug-Drug Interaction of Non-potassium-Sparing Diuretics and Regular Laxative Use

Signs for a potential drug-drug interaction of non-potassium-sparing diuretics and concurrent regular laxative use were observed. However, tests for interaction were not statistically significant. This can mainly be explained by the low statistical power of our analysis because exposure to concurrent use of both drug classes was rare (1.3% and 1.6% among antihypertensive drug users in the ESTHER study and UK Biobank, respectively). The likely underestimation of the cardiovascular risk of regular laxative users due to the previously discussed healthy-user/sick-stopper bias and a prevalent user design will have limited the chance for a detection of a statistically significant interaction in our study.

### Strengths and Limitations

The limitations of our study and their potential impact on the study results have been discussed earlier and include the prevalent user design, no repeated drug assessment, and a limited extent to control for confounding (in particular for the severity of heart failure and CHD). Furthermore, serum potassium measurements were not available from the two analyzed cohort studies. In addition to information on potassium-influencing drug use, such measurements would have been quite informative to provide evidence that the increased CVM observed in our study is indeed related to a drug-induced electrolyte disorder. However, this is already evident from previous cohort studies, which we summarized in a systematic review about potassium measurements and cardiovascular outcomes [14]. The included study of Cohen and colleagues, for instance, observed a 2.6-fold increased risk for a composite cardiovascular outcome in diuretic-treated hypertensive patients with low serum potassium levels compared to individuals with adequate potassium levels [11]. Furthermore, the risk of hypokalemia by chronic laxative use has well been documented by Xing et al. [24] and Kokot et al. [23].

This is the first investigation about the concurrent use of non-potassium-sparing diuretics and laxatives, which was only feasible due to a thorough medication assessment of OTC drugs, which are not available in claims databases. Another strength of our study is that analyses followed the same protocol in two large cohort studies with a long follow-up for CVM. Thus, statistically significant results from a derivation cohort (ESTHER) were confirmed in a replication cohort (UK Biobank). In addition, meta-analyses of the two studies were conducted to increase the statistical power. However, the pooling of the two studies is debatable because they originate from different countries with different drug prescribing patterns and their data assessment methods varied. We addressed these concerns by deriving harmonized variable definitions and by using identical statistical methods in both cohorts along with conservative random-effects meta-analyses, which consider between-study heterogeneity. No signs of statistical heterogeneity were observed in any of the meta-analyses (Cochrane’s *Q* test’s *p* > 0.05), which ensured us that it was appropriate to conduct the meta-analyses.

## Conclusion

This analysis in two large cohort studies yielded consistent results with respect to an association of diuretics use overall, but not regular laxative use, with CVM among older adults treated with antihypertensive drugs. Signs for a drug-drug interaction of non-potassium-sparing diuretics and laxatives were detected. Interactions, however, were not statistically significant, mainly because concurrent use was rare in the two studies. Nevertheless, we observed a statistically significant 2-fold increased mortality in concurrent users of non-potassium-sparing diuretics and laxatives. Therefore, we would recommend physicians to clarify additional laxative use in their patients who receive non-potassium-sparing diuretics, inform them about the cardiovascular risk of concurrent use of these drug classes, and monitor serum potassium levels in shorter intervals in patients that use laxatives on a regular basis. Of course, serum potassium levels are being routinely checked in clinical practice, but maybe the risk of additional self-medication has been underestimated in clinical practice before. This is why we think that a closer monitoring with shorter intervals (e.g., every 3 months) could provide an opportunity for cardiovascular prevention. However, before implementing in clinical routine, clinical trials are needed to evaluate if tighter potassium monitoring intervals have an advantage for concurrent users. Furthermore, pharmacists should be vigilant if patients regularly purchase laxatives in a pharmacy and make them aware of the potential drug-drug interaction between non-potassium-sparing diuretics and laxatives.

## Electronic Supplementary Material

ESM 1 (PDF 234 kb)
