# Supplementary material for: The Associations of Diuretics and Laxatives Use with Cardiovascular Mortality. An Individual Patient-Data Meta-analysis of Two Large Cohort Studies
Source: Cardiovasc Drugs Ther. 2019 Aug 2;33(5):567–79. doi: 10.1007/s10557-019-06894-w (PMC6904395; doi:10.1007/s10557-019-06894-w)
Supplement: Supplementary file 1 — (PDF 234 kb) [file 10557_2019_6894_MOESM1_ESM.pdf]

Electronic Supplementary Material (ESM 1)

Cardiovascular Drugs and Therapy
https://doi.org/10.1007/s10557-019-06894-w

Article

The associations of diuretics and laxatives use with cardiovascular mortality. An individual patient-data meta-analysis of two large cohort studies

Liesa Katharina Hoppe, Dana Clarissa Muhlack, Wolfgang Koenig, Hermann Brenner, Ben Schöttker

Table of Contents

Table S1. Use of antihypertensive drug classes at baseline in the ESTHER study and the UK Biobank 2

Table S2. Baseline characteristics of laxative users compared to non-users of laxatives of the analyzed participants with antihypertensive treatment in the ESTHER study and the UK Biobank 3

Table S3. Baseline characteristics of diuretics users compared to non-users of diuretics of the analyzed participants with antihypertensive treatment in the ESTHER study and the UK Biobank .. 5

Table S4. Baseline characteristics of users of non-potassium-sparing diuretics compared to users of potassium-sparing diuretics / combinations of non-potassium-sparing diuretics with potassium or potassium-sparing diuretics of the analyzed participants with antihypertensive treatment in the ESTHER study and the UK Biobank 7

Table S5. Associations with CVM in users of potassium-sparing diuretics / combinations of non-potassium-sparing diuretics with potassium or potassium-sparing diuretics compared to non-users of diuretics in the UK Biobank 9

Table S6. Associations with CVM comparing users and non-users of laxatives, diuretics overall, and diuretics in specific in the ESTHER study using only the first 7 years of follow-up or the complete follow-up of 14 years 10

Table S1. Use of antihypertensive drug classes at baseline in the ESTHER study and the UK Biobank

**Drug class**	**ESTHER** (n = 4,253)		**UK BIOBANK** (n = 105,359)	
	**n**	**(%)**	**n**	**(%)**
Diuretics	897	(21.1)	38,227	(36.3)
β-blockers	2,095	(49.3)	32,193	(30.6)
Calcium channel blockers	1,168	(27.5)	33,463	(31.7)
Angiotensin-converting enzyme inhibitors	1,660	(39.0)	46,001	(43.7)
Angiotensin receptor blockers	513	(12.1)	19,963	(19.0)

UK, United Kingdom.

Table S2. Baseline characteristics of laxative users compared to non-users of laxatives of the analyzed participants with antihypertensive treatment in the ESTHER study and the UK Biobank

	**ESTHER** (n = 4,253)			**UK BIOBANK** (n = 105,359)		
**Laxative use**	**Yes** (n = 345)	**No** (n = 3,908)		**Yes** (n = 4,322)	**No** (n = 101,037)	
**Characteristics**	**n [%]**	**n [%]**	**p-value**	**n [%]**	**n [%]**	**p-value**
Age ≥ 65 years	57.1	46.8	< **0.001**	41.6	36.0	< **0.001**
Sex (male)	25.8	46.8	< **0.001**	32.3	53.8	< **0.001**
Smoking			0.181			< **0.001**
Never	54.8	51.5		46.1	48.1	
Former	30.4	35.3		42.6	43.1	
Current	14.9	13.2		11.4	8.8	
Vigorous physical activity [a]			0.218			< **0.001**
No	61.1	57.6		57.5	45.3	
Yes	39.0	42.4		42.6	54.7	
Alcohol consumption [b]			< **0.001**			< **0.001**
Abstainer	48.0	35.3		22.5	11.7	
WHO category I	47.3	58.6		57.3	63.0	
WHO category II/III	4.7	6.1		20.2	25.3	
SBP (mmHg)			0.176			< **0.001**
< 140	34.3	32.4		44.2	35.9	
140 to < 160	44.3	41.6		36.4	39.6	
≥ 160	21.4	26.0		19.4	24.6	
BMI (kg/m^2)			0.060			< **0.001**
< 25	20.6	17.9		20.8	17.9	
25 to < 30	40.9	47.5		38.2	42.6	
≥ 30	38.6	34.6		41.0	39.6	
Urinary albumin (mg /L)			0.575			0.615
< 20	79.5	78.2		66.9	66.3	
≥ 20	20.5	21.8		33.2	33.8	
Diabetes mellitus	26.3	22.2	0.080	10.9	10.8	0.788
Heart failure [c]	25.4	17.1	< **0.001**	N.A.	N.A.	N.A.
CHD	25.5	22.0	0.129	21.3	16.6	< **0.001**
History of MI	12.6	10.0	0.138	9.6	8.9	0.130
History of stroke	8.9	5.2	**0.005**	3.9	2.8	< **0.001**
Anticholinergic drug use	9.0	4.9	**0.001**	18.5	6.3	< **0.001**
Opioid use	2.0	0.7	**0.010**	26.1	8.4	< **0.001**
Diuretics use	29.6	20.3	< **0.001**	43.6	36.0	< **0.001**
Non-potassium-sparing diuretics	15.4	10.3	**0.004**	39.3	33.8	< **0.001**
Potassium-sparing diuretics or combinations [d]	14.2	10.1	**0.016**	4.3	2.2	< **0.001**

Bold printed: statistically significant ($p < 0.05$).

[a] Vigorous physical activity was measured in hours per week (ESTHER study) and in number of days per week of at least 10 min of activity (UK Biobank): “No”: Participants not doing any amount of vigorous physical activity, “Yes”: Participants doing any amount of vigorous physical activity.

[b] Alcohol consumption in categories of the WHO: Category I including women with an alcohol consumption of 0-19.99 g/day or men with 0-39.99 g/day, category II including women with an alcohol consumption of 20-39.99 g/day or men with 40-59.99 g/day, and category III including women with an alcohol consumption of ≥ 40 g/day or men with ≥ 60 g/day.

[c] Not sufficiently assessed in the UK Biobank (unreliable self-report) and therefore not applicable for use.

[d] Group comprises users of potassium-sparing diuretics / combinations of non-potassium-sparing diuretics with potassium or potassium-sparing diuretics.

BMI, Body Mass Index; *CHD*, Coronary heart disease; *MI*, Myocardial infarction; *N.A.*, Not applicable; *SBP*, Systolic blood pressure; *SD*, Standard deviation; *UK*, United Kingdom; *WHO*, World health organization.

Table S3. Baseline characteristics of diuretics users compared to non-users of diuretics of the analyzed participants with antihypertensive treatment in the ESTHER study and the UK Biobank

**Diuretics use**	**ESTHER** (n = 4,253)			**UK BIOBANK** (n = 105,359)		
	**Yes** (n = 897)	**No** (n = 3,356)		**Yes** (n = 38,227)	**No** (n = 67,132)	
**Characteristics**	**n [%]**	**n [%]**	**p-value**	**n [%]**	**n [%]**	**p-value**
Age ≥ 65 years	56.6	45.2	< **0.001**	38.9	34.8	< **0.001**
Sex (male)	40.5	46.3	**0.002**	42.7	58.7	< **0.001**
Smoking			0.251			< **0.001**
Never	54.0	51.2		49.2	47.4	
Former	34.0	35.1		42.2	43.6	
Current	12.1	13.7		8.6	9.0	
Vigorous physical activity [a]			0.094			< **0.001**
No	60.4	57.3		48.7	44.2	
Yes	39.6	42.8		51.4	55.8	
Alcohol consumption [b]			< **0.001**			< **0.001**
Abstainer	42.6	34.7		13.6	11.4	
WHO category I	53.7	58.8		61.2	63.6	
WHO category II/III	3.7	6.5		25.2	25.0	
SBP (mmHg)			0.797			< **0.001**
< 140	33.4	32.3		34.9	36.9	
140 to < 160	41.0	42.1		40.2	39.0	
≥ 160	25.6	25.6		25.0	24.1	
BMI (kg/m^2)			< **0.001**			< **0.001**
< 25	12.7	19.6		15.1	19.6	
25 to < 30	40.9	48.6		39.5	44.0	
≥ 30	46.4	31.8		45.4	36.4	
Urinary albumin (mg /L)			**0.007**			< **0.001**
< 20	75.0	79.2		67.4	65.7	
≥ 20	25.1	20.8		32.6	34.3	
Diabetes mellitus	30.9	20.3	< **0.001**	10.2	11.2	< **0.001**
Heart failure [c]	28.5	15.0	< **0.001**	N.A.	N.A.	N.A.
CHD	31.2	19.9	< **0.001**	11.0	20.1	< **0.001**
History of MI	15.4	8.9	< **0.001**	5.8	10.7	< **0.001**
History of stroke	8.2	4.7	< **0.001**	3.2	2.6	< **0.001**
Anticholinergic drug use	5.5	5.2	0.768	7.4	6.5	< **0.001**
Opioid use	1.6	0.6	**0.006**	11.1	8.0	< **0.001**
Laxative use	11.4	7.2	< **0.001**	4.9	3.6	< **0.001**

Bold printed: statistically significant ($p < 0.05$)

[a] Vigorous physical activity was measured in hours per week (ESTHER study) and in number of days per week of at least 10 min of activity (UK Biobank): "No": Participants not doing any amount of vigorous physical activity, "Yes": Participants doing any amount of vigorous physical activity.

[b] Alcohol consumption in categories of the WHO: Category I including women with an alcohol consumption of 0-19.99 g/day or men with 0-39.99 g/day, category II including women with an alcohol consumption of 20-39.99 g/day or men with 40-59.99 g/day, and category III including women with an alcohol consumption of ≥ 40 g/day or men with ≥ 60 g/day.

[c] Not sufficiently assessed in the UK Biobank (unreliable self-report) and therefore not applicable for use.

BMI, Body Mass Index; *CHD*, Coronary heart disease; *MI*, Myocardial infarction; *N.A.*, Not applicable; *SBP*, Systolic blood pressure; *SD*, Standard deviation; *UK*, United Kingdom; *WHO*, World health organization.

Table S4. Baseline characteristics of users of non-potassium-sparing diuretics compared to users of potassium-sparing diuretics / combinations of non-potassium-sparing diuretics with potassium or potassium-sparing diuretics of the analyzed participants with antihypertensive treatment in the ESTHER study and the UK Biobank

	**ESTHER** (n = 4,253)			**UK BIOBANK** (n = 105,359)		
**Users of**	**Non-potassium-sparing diuretics** (n = 455)	**Potassium-sparing diuretics or diuretics combinations** [a] (n = 442)		**Non-potassium-sparing diuretics** (n = 35,821)	**Potassium-sparing diuretics or diuretics combinations** [a] (n = 2,406)	
**Characteristics**	**n [%]**	**n [%]**	**p-value**	**n [%]**	**n [%]**	**p-value**
Age ≥ 65 years	58.2	55.0	0.324	38.8	40.4	0.112
Sex (male)	43.7	37.1	0.043	42.8	41.0	0.074
Smoking			0.054			< **0.001**
Never	52.6	55.4		49.5	45.6	
Former	37.2	30.6		42.1	43.9	
Current	10.1	14.0		8.5	10.5	
Vigorous physical activity [b]			0.976			< **0.001**
No	60.3	60.4		47.9	59.4	
Yes	39.7	39.6		52.1	40.6	
Alcohol consumption [c]			0.880			< **0.001**
Abstainer	41.7	43.4		13.1	20.9	
WHO category I	54.5	53.0		61.5	56.5	
WHO category II/III	3.8	3.6		25.4	22.7	
SBP (mmHg)			**0.013**			< **0.001**
< 140	29.0	38.0		33.8	51.0	
140 to < 160	42.7	39.2		40.6	33.2	
≥ 160	28.3	22.8		25.6	15.8	
BMI (kg/m^2)			0.479			< **0.001**
< 25	12.1	13.4		15.1	15.7	
25 to < 30	39.6	42.3		39.8	34.5	
≥ 30	48.4	44.3		45.1	49.9	

	ESTHER (n = 4,253)			UK BIOBANK (n = 105,359)		
**Users of**	**Non-potassium-sparing diuretics** (n = 455)	**Potassium-sparing diuretics or diuretics combinations [a]** (n = 442)		**Non-potassium-sparing diuretics** (n = 35,821)	**Potassium-sparing diuretics or diuretics combinations [a]** (n = 2,406)	
**Characteristics**	**n [%]**	**n [%]**	**p-value**	**n [%]**	**n [%]**	**p-value**
Urinary albumin (mg /L)			**0.002**			0.124
< 20	70.4	79.6		67.6	65.1	
≥ 20	29.6	20.4		32.4	34.9	
Diabetes mellitus	35.3	26.3	**0.004**	10.0	12.7	**< 0.001**
Heart failure [d]	31.5	25.4	**0.046**	N.A.	N.A.	N.A.
CHD	35.2	27.2	**0.010**	10.1	24.0	**< 0.001**
History of MI	17.2	13.4	0.119	5.1	15.5	**< 0.001**
History of stroke	9.0	7.4	0.393	3.2	3.5	0.305
Anticholinergic drug use	4.8	6.1	0.401	7.2	10.6	**< 0.001**
Opioid use	1.3	1.8	0.553	10.7	17.1	**< 0.001**
Laxative use	11.7	11.1	0.791	4.7	7.8	**< 0.001**

Bold printed: statistically significant ($p < 0.05$).

[a] Group comprises users of potassium-sparing diuretics / combinations of non-potassium-sparing diuretics with potassium or potassium-sparing diuretics.

[b] Vigorous physical activity was measured in hours per week (ESTHER study) and in number of days per week of at least 10 min of activity (UK Biobank): “No”: Participants not doing any amount of vigorous physical activity, “Yes”: Participants doing any amount of vigorous physical activity.

[c] Alcohol consumption in categories of the WHO: Category I including women with an alcohol consumption of 0-19.99 g/day or men with 0-39.99 g/day, category II including women with an alcohol consumption of 20-39.99 g/day or men with 40-59.99 g/day, and category III including women with an alcohol consumption of ≥ 40 g/day or men with ≥ 60 g/day.

[d] Not sufficiently assessed in the UK Biobank (unreliable self-report) and therefore not applicable for use.

BMI, Body Mass Index; *CHD*, Coronary heart disease; *MI*, Myocardial infarction; *N.A.*, Not applicable; *SBP*, Systolic blood pressure; *UK*, United Kingdom; *WHO*, World Health Organization.

Table S5. Associations with CVM in users of potassium-sparing diuretics / combinations of non-potassium-sparing diuretics with potassium or potassium-sparing diuretics compared to non-users of diuretics in the UK Biobank

**Drug class**	**UK BIOBANK** (n = 105,359; 1,616 cases)		
	**n** [a]	**n_{cases}** [b]	**HR (95%CI)** [c]
Non-users of diuretics	67,132	887	Ref.
Users of potassium-sparing diuretics	490	26	**2.76 (1.87; 4.07)**
Users of non-potassium-sparing diuretics combined with potassium-sparing diuretics	1,675	101	**3.15 (2.56; 3.87)**
Users of non-potassium-sparing diuretics combined with potassium	241	5	1.51 (0.63; 3.62)

Bold printed: statistically significant ($p < 0.05$).

[a] Sample sizes exemplarily taken from imputed data set no. 1.

[b] Case numbers do not add up to the total study case number (n=1,616) due to 597 cases within the users of non-potassium-sparing diuretics (not shown).

[c] Adjusted for age, sex, smoking status, physical activity, alcohol consumption, systolic blood pressure, body mass index, potential kidney damage (urinary albumin ≥ 20 mg/L), diabetes mellitus, coronary heart disease, history of myocardial infarction, history of stroke, anticholinergic drug use, and use of opioids.

CI, Confidence interval; *CVM*, Cardiovascular mortality; *HR*, Hazard ratio; *Ref.*, Reference; *UK*, United Kingdom.

Table S6. Associations with CVM comparing users and non-users of laxatives, diuretics overall, and diuretics in specific in the ESTHER study using only the first 7 years of follow-up or the complete follow-up of 14 years

**Drug class**	**ESTHER (7 years of FUP)** (n = 4,253; 169 cases)			**ESTHER (14 years of FUP)** (n = 4,253; 476 cases)		
	**n [a]**	**n_{cases}**	**HR (95%CI)[b]**	**n [a]**	**n_{cases}**	**HR (95%CI) [b]**
**Laxatives**						
Non-users	3,890	153	Ref.	3,890	433	Ref.
Users	363	16	1.09 (0.64; 1.87)	363	43	0.99 (0.70; 1.41)
**Diuretics overall**						
Non-users	3,356	109	Ref.	3,356	327	Ref.
Users	897	60	**1.45 (1.06; 2.06)**	897	149	**1.39 (1.13; 1.70)**
**Diuretics in specific**						
Non-users	3,356	109	Ref.	3,356	327	Ref.
Users of non-potassium-sparing diuretics	455	40	**1.75 (1.21; 2.53)**	455	90	**1.49 (1.17; 1.89)**
Users of potassium-sparing diuretics or combinations [c]	442	20	0.96 (0.59; 1.54)	442	59	1.10 (0.83; 1.46)

Bold printed: statistically significant ($p < 0.05$).

[a] Sample sizes exemplarily taken from imputed data set no. 1.

[b] Adjusted for age, sex, smoking status, physical activity, alcohol consumption, systolic blood pressure, body mass index, potential kidney damage (urinary albumin ≥ 20 mg/L), diabetes mellitus, heart failure, coronary heart disease, history of myocardial infarction, history of stroke, anticholinergic drug use, and use of opioids.

[c] Group comprises users of potassium-sparing diuretics / combinations of non-potassium-sparing diuretics with potassium or potassium-sparing diuretics.

CI, Confidence interval; *CVM*, Cardiovascular mortality; *FUP*, Follow-up; *HR*, Hazard ratio; *Ref.*; Reference.
